# How Many Concussions Would It Take for Athletes to Choose to Discontinue Participation in Their Primary Sport?

**DOI:** 10.3390/ijerph18041582

**Published:** 2021-02-08

**Authors:** Matthew R. Monaco, Britton W. Brewer, Judy L. Van Raalte, Christine N. May

**Affiliations:** 1 Department of Psychology, Springfield College, 263 Alden Street, Springfield, MA 01109, USA; mmonaco@springfieldcollege.edu (M.R.M.); jvanraal@springfieldcollege.edu (J.L.V.R.); christinemay811@gmail.com (C.N.M.); 2 College of Health Sciences, Wuhan Sports University, 461 Luoyu Road, Wuhan 430079, China

**Keywords:** injury risk, sport ethic, athletic identity

## Abstract

The process by which athletes decide to continue or discontinue sport participation after concussion has not been explicated. Intercollegiate and club sport athletes (*N* = 394) completed an online survey that included assessments of demographic factors, the total number of concussions (and anterior cruciate ligament (ACL) tears) that would prompt sport retirement, concussion history, and athletic identity. On average, participants reported that they would retire from their primary sport after sustaining 3 to 4 concussions (and approximately 2 ACL tears). The total number of concussions reported was negatively correlated with the number of additional concussions it would take to precipitate sport retirement. Athletic identity was positively associated with the number of concussions that participants with a history of one or more concussions reported would prompt them to retire from their primary sport. The results provide information of potential utility to professionals implementing concussion education programs and working with athletes at risk of experiencing concussion.

## 1. Introduction

Sport-related concussion has emerged as an important public health concern, with an estimated 1.7 to 3 million sport- and recreation-related concussions sustained each year by individuals in the United States alone [1]. After experiencing a concussion, athletes may exhibit signs such as loss of consciousness, amnesia, disorientation, nausea, and problems with vision, balance, and motor coordination [2]. Post-traumatic symptoms reported by athletes after concussion include concentration difficulties, dizziness, drowsiness, fatigue, and mental fogginess [3]. Cognitive and neuromotor impairments experienced after sport-related concussion typically persist for 1 to 4 weeks, depending on the age and sex of the athlete and domain of impairment under consideration [4,5]. There appears to be a cumulative effect of concussions on the post-injury consequences encountered by athletes such that “individuals with a history of multiple concussions are at an increased risk for experiencing more signs and symptoms following a concussion, as well as longer symptom duration and prolonged recovery” [6] (p. 9). In addition, concussions elevate the risk of subsequent concussive [7,8] and musculoskeletal [9] injuries, and multiple concussions are associated with a heightened long-term risk for depression [8,10].

In light of the perceived [11] and actual [6,7,8,9,10] risks associated with multiple concussions as well as recent improvements in athlete awareness and expansion of educational programming regarding sport-related concussion [12], athletes may be confronted with the question of when or if they should discontinue participation in their primary sport. Sports healthcare professionals can offer athletes guidance on how to proceed after sustaining multiple concussions, but athletes may fail to disclose concussions or concussion-related symptoms to medical professionals [13,14] and ultimately may have to answer for themselves the question “How many (or how many more) concussions would it take for me to retire?” Although knowledge of such a number (which, for the sake of brevity, we have labeled “concussion retirement number”) could be of considerable practical utility in, for example, evaluating athletes’ risk tolerance or motivation to engage in preventive behavior, the topic has not been examined systematically.

One factor that may influence the concussion retirement number is athletic identity, which refers as the extent to which individuals identify with the athlete role [15]. According to Hughes and Coakley [16], people who self-identify strongly and exclusively as athletes tend to subscribe to the sociocultural “sport ethic” value system that emphasizes the importance of athletes making sacrifices for their sport, accepting risks, and playing through pain and injury. In support of this claim, positive associations have been documented between athletic identity and the tendencies to report playing through pain [17] and over-adhering to sport injury rehabilitation [18]. Further, in a study of male intercollegiate hockey players, athletic identity was found to moderate the association between subjective norms and concussion symptom reporting such that “for any given level of perceived reporting norms, higher athletic identity will result in slightly greater odds of under reporting behavior” [19] (p. 98).

In the absence of research on how many concussions it would take for athletes to discontinue participation in their primary sport (i.e., concussion retirement number), the primary purpose of the current study was to obtain preliminary descriptive data in that regard. For the sake of comparison with another serious sport injury, data were also collected on the retirement number for anterior cruciate ligament (ACL) tears (i.e., ACL retirement number). In addition to involving a different body location than concussions, ACL tears are typically treated surgically and have a considerably longer period of recovery and rehabilitation (i.e., ≥6 months) [20]. A secondary purpose was to examine the relationship between athletic identity and the concussion retirement number. Based on the theoretical position of Hughes and Coakley [16] and the findings of Weinberg et al. [17], Hilliard et al. [18], and Kroshus et al. [19], it was hypothesized that athletic identity would be positively associated with the concussion retirement number.

## 2. Materials and Methods

### 2.1. Participants

Participants were 394 intercollegiate and club sport athletes (199 men and 195 women) who responded to a survey question inquiring as to whether they had ever experienced a concussion. Participants were from basketball (*n* = 17), baseball (*n* = 32), cheerleading (*n* = 3), cross country (*n* = 11), cross fit (*n* = 1), dance (*n* = 10), diving (*n* = 2), equestrian (*n* = 2), field hockey (*n* = 20), figure skating (*n* = 1), American football (*n* = 73), golf (*n* = 8), gymnastics (*n* = 21), ice hockey (*n* = 3), lacrosse (*n* = 26), rugby (*n* = 32), running (*n* = 1), ski and snowboarding (*n* = 1), soccer (*n* = 44), softball (*n* = 5), swimming (*n* = 16), track and field (*n* = 41), volleyball (*n* = 24), and wrestling (*n* = 2) school teams across the United States. The average age of participants was 19.77 (*SD* = 1.57, range = 18 to 33) years, with the majority of student-athletes being first or second-year students. The sample self-identified as 88 percent White, 7 percent African American, 4 percent Asian, and 1 percent other.

### 2.2. Instruments

An online survey on the Qualtrics platform was used to collect data pertaining to demographics, athletic identity, the total number of concussions and ACL tears it would take for athletes to decide to retire from their primary sport (i.e., concussion and ACL retirement numbers, respectively), and medical history of concussions and ACL tears. Demographic factors assessed included age, gender, year in college, ethnicity, race, and primary sport. The Athletic Identity Measurement Scale (AIMS) [15,21], which consists of 7 items that are scored on a 7-point Likert-type scale with endpoints of “strongly disagree” and “strongly agree,” was used to assess athletic identity. “I consider myself an athlete” and “Sport is the most important part of my life” are examples of AIMS items. Evidence in support of the reliability and validity of the AIMS has been obtained [15,21]. A Cronbach’s alpha coefficient of 0.82 was found for the AIMS in the current study. In addition to questions asking, “How many concussions would you have to sustain for you to retire from (primary sport)?” and “How many ACL tears you would have to sustain for you to retire from (primary sport)?”, participants were asked to indicate their medical history of ACL tears and diagnosed and undiagnosed concussions. 

### 2.3. Procedure

After receiving Springfield College Institutional Review Board approval, three approaches to recruit participants were implemented: (a) members of 13 intercollegiate or club sport teams at a small college and 1 to 5 intercollegiate or club sport teams at 7 universities were contacted via email or text and invited to participate in the study; (b) invitations to participate in the study were posted on social media sites such as Facebook, Twitter, Instagram, LinkedIn, and Reddit; and (c) students enrolled in Introduction to Psychology classes at a small college were invited to participate in the study through an online posting. In the email to prospective participants and social media postings, a two-sentence overview of the study was provided along with the URL link to the survey on Qualtrics. An informed consent statement was included in the introduction to the study. Participants were directed out of the survey and excluded from the study if they: (a) did not consent to be in the study; (b) indicated that they were under the age of 18; or (c) reported that they were not an active participant on an intercollegiate or club sports roster. 

### 2.4. Data Analysis

Quantitative data were entered into the IBM Statistical Package for the Social Sciences (SPSS) version 24. Data were screened for accuracy and completeness. An adjusted concussion retirement number was calculated by subtracting participants’ total number of concussions (summed across diagnosed, sport-related, and non-sport-related concussions) reported from their concussion retirement number. For example, if an athlete reported having experienced a total of 2 concussions and said it would take 4 concussions to cease primary sport participation, the adjusted concussion retirement number would be 2. This “How many (or how many more) concussions?” process was also done for ACL tears. Descriptive statistics were computed for the primary variables of interest. An independent-samples t-test was conducted to compare the adjusted concussion retirement numbers of participants who reported having experienced no concussions with those who reported having experienced one or more concussions. A Pearson correlation was computed to examine the association between the total number of concussions reported and the adjusted concussion retirement number.

Paired-samples t-tests were conducted to assess the differences between the concussion retirement and adjusted concussion retirement numbers and between the ACL retirement and adjusted ACL retirement numbers. We interpreted Cohen’s *d* such that 0.2 represented a small effect size, 0.5 represented a medium effect size, and 0.8 represented a large effect size [22]. A hierarchical multiple regression analysis was conducted to predict the concussion retirement number. Concussion history (represented by a dummy vector in which participants reporting no history of concussions were coded with a 0 and participants reporting a history of one or more concussions were coded with a 1), AIMS scores, and an interaction vector representing the product of the dummy vector and AIMS scores were entered on the first, second, and third steps of the analysis, respectively.

## 3. Results

Theoretically possible values for the concussion retirement number in light of participants’ self-reported concussion history and continued sport involvement (i.e., values > the total number of concussions reported) were obtained from 299 participants, whose data were subjected to further analysis. Descriptive statistics for the main variables of interest are presented in Table 1. Nearly half (49%) of the participants (*n* = 146) reported having sustained at least one concussion. Thirteen participants (4%) reported having experienced at least 1 ACL tear.

Results of the independent-samples t-test indicated that the mean adjusted concussion retirement number for participants who reported having experienced no concussions (*M* = 3.53, *SD* = 1.39) was significantly higher than that for participants who reported having experienced one or more concussions (*M* = 3.11, *SD* = 1.92), *t*(264.30) = 2.13, *p* = 0.03, *d* = 0.25. The Pearson correlation between the total number of concussions reported and the adjusted concussion retirement number was statistically significant, *r* = −0.14, *p* = 0.01. Results of the paired-samples t-tests showed that the concussion retirement number and adjusted concussion retirement number were significantly higher than the ACL retirement number and adjusted ACL retirement number, *t*(293) = 18.12, *p* < 0.001, *d* = 0.88, and *t*(293) = 12.76, *p* < 0.001, *d* = 0.63, respectively.

In the first step of the hierarchical regression analysis (see Table 2), concussion history was positively associated with concussion retirement number, *R*^2^ = 0.08, *F*(1, 297) = 24.93, *p* < 0.001. The contribution of athletic identity to the prediction of the criterion variable attained statistical significance, Δ*R*^2^ = 0.03, *F*(1, 296) = 9.06, *p* = 0.003, and the interaction between concussion history and athletic identity accounted for a significant increment in explained variance, Δ*R*^2^ = 0.02, *F*(1, 295) = 7.71, *p* = 0.006. The regression equation for the full model was statistically significant, *R*^2^ = 0.13, *F*(3, 295) = 14.39, *p* < 0.001. The nature of the significant interaction between concussion history and athletic identity was such that athletic identity was positively associated with concussion retirement number (*r* = 0.35, *p* < 0.001) for participants who reported having experienced one or more concussions and not significantly associated with concussion retirement number (*r* = 0.03, *p* = 0.67) for participants who reported having experienced no concussions.

## 4. Discussion

The main purpose of the study was to obtain a preliminary assessment of the number of concussions that would prompt athletes to retire from their primary sport. Consistent with the sport ethic [16], in which athletes readily accept risks to their health and safety for the sake of sport participation, athletes reported a willingness to experience approximately 3 to 4 concussions before retiring voluntarily from their primary sport. This finding is both noteworthy and concerning in that Guskiewicz et al. [10] found that retired professional football players who were diagnosed with 3 or more concussions were three times more likely than retired players with no concussions to be diagnosed with depression.

Although the significant negative correlation between self-reported number of concussions sustained and adjusted total number of concussions indicated to precipitate retirement suggests that the participants took their concussion history into account when selecting a concussion retirement number, the association is weak. Compared to the athletes in this study who reported having no concussions, athletes who reported a history of one or more concussions had an adjusted concussion retirement number that was significantly—but less than half a concussion—lower. The participants, therefore, appeared to “kick the can down the road” and defer retirement until they have sustained 2 to 3 more concussions than they have already experienced.

The significantly higher values for the retirement and adjusted retirement numbers for concussions than for ACL tears indicates that participants made a clear distinction between the two types of injuries. The reasons for the distinction, however, are unclear. Possible sources of the distinction include greater perceived severity, a longer rehabilitation period, a greater likelihood of surgical intervention, and a more adverse impact on sport performance for ACL tears than for concussions. It is also possible that concussions continue to be perceived as less serious (e.g., “getting your bell rung”) than other major sport injuries such as ACL tears.

With respect to the secondary purpose of the current study—to examine the relationship between athletic identity and the concussion retirement number—the hypothesized positive association between the two variables was found only among athletes with a self-reported history of 1 or more previous concussions. For participants with no self-reported history of concussions, athletic identity was not significantly related to the concussion retirement number. It is therefore possible that the role played by athletic identity in the process in deciding how to act in the face of sport-related risk increases as the actual risks encountered by athletes (as indicated by history of previous concussions) accumulate. Among participants reporting one or more previous concussions, the apparent willingness of those who expressed strong identification with the athlete role to experience more concussions before retiring from their primary sport than those who expressed weak(er) identification with the athlete role is noteworthy in light of research documenting positive associations between athletic identity and multiple injury occurrence [23], postinjury emotional disturbance [15,24,25], and, of particular relevance to the current study, severity of concussion symptoms [26]. The extent to which the potential influence of athletic identity on the decision of athletes to cease participation in their primary sport overlaps with the value preferences of athletes for stimulation [27] merits consideration in future research.

Several limitations should be considered when interpreting results of the study and planning subsequent research on the topic. First, as a consequence of collecting data online, issues with Wi-Fi resulted in incomplete data for some participants (*n* = 80). Second, the data were collected exclusively through self-report in a cross-sectional design. Beyond face validity, the psychometric properties of athletes’ estimates of how many concussions (and ACL tears) it would take for them to retire from their primary sport are not known. The temporal stability of the estimates could be ascertained in future research by repeated administration of the pertinent items over a brief period of time. The validity of the estimates could be evaluated by examining longitudinally the extent to which athletes’ estimates of how many concussions (and ACL tears) it would take for them to retire from their primary sport correspond to their actual decisions to continue or discontinue sport participation after incurring concussion(s) (and ACL tears). Any concerns about the accuracy of participants’ self-reported (diagnosed) concussions could be addressed in future research through examination of medical records. Third, due to the convenience sampling method used in the current study, the participants were relatively homogeneous with respect to age, race, and institutional affiliation. Similarly, because data were not collected from a nationally representative sample, the findings may not generalize to the general athlete population. There may also have been selection bias such that athletes with a history of concussions may have been more likely to respond to the survey. Future research with more diverse and representative samples is needed to determine the generalizability of the current findings. Fourth, given the preliminary nature of the current study, no information was collected pertaining to the rationale underlying participants’ concussion retirement numbers. Open-ended questions could be added to the survey in future studies to determine the extent to which factors such as recommendations from medical professionals, vicarious experiences of concussions sustained by others (e.g., teammates, professional athletes), and sociocultural norms might contribute to the quantitative data provided by athletes.

## 5. Conclusions

The preliminary estimates of how many concussions it would take athletes to discontinue participation in their primary sport obtained in the current study findings have several important implications for application. First, the results reaffirm the high levels of risk to health and wellbeing that athletes are willing to experience in pursuit of their sport participation goals (Hughes and Coakley, 1991) and the connection of athletic identity to that risk (Brewer et al., 1993). Knowing where athletes stand in terms of risk acceptance for concussions and athletic identity may assist in the interpreting their psychological responses to concussion and motivation to return to sport. Second, assessing how many additional concussions athletes would be willing to incur before discontinuing participation in their primary sport can serve as a point of departure in discussing the risks of continued sport involvement with athletes after concussion from a probabilistic, research-based perspective. Third, the concussion retirement number can be ascertained before and after concussion education interventions as a means of determining the impact of the interventions in a practical, personally relevant way. Enhanced knowledge about sport-related concussion has limited utility if not accompanied by a change in intended response to risk. Ultimately, understanding how athletes plan to proceed in response to sport injuries they might experience can enable sport rehabilitation professionals to better serve the athletes with whom they work.

## Figures and Tables

**Table 1:** Descriptive statistics for main outcome measures.

Variable	Mode	Median	*M*	*SD*
Total number of concussions to precipitate	3	3.50	4.04	1.85
retirement				
Adjusted number of concussions to precipitate	3	3	3.33	1.68
retirement				
Total number of concussions reported	0	0	0.71	1.05
Total number of ACL tears to precipitate	2	2	2.11	1.02
retirement *				
Adjusted number of ACL tears to	2	2	2.05	1.02
precipitate retirement *				
Total number of ACL tears reported *	0	0	0.06	0.28
AIMS	43	39	38.43	6.33

Note. *n* = 299 except as indicated by *, where *n* = 294. ACL = anterior cruciate ligament; AIMS = Athletic Identity Measurement Scale.

**Table 2:** Hierarchical regression analysis predicting concussion retirement number.

Predictor	Δ*R*^2^	R^2^	β	*F*	*F*_Change_
Step 1—Concussion history	0.08	0.08	0.28	24.93 **	24.93 **
Step 2—AIMS	0.03	0.11	0.17	17.33 **	9.06 *
Step 3—Concussion history	0.02	0.13	0.95	14.39 **	7.71 *
x AIMS interaction					

Note. *n* = 299. AIMS = Athletic Identity Measurement Scale. ** *p* < 0.001; * *p* < 0.01.

## Data Availability

The data presented in this study are available on request from the corresponding author.
